# Association between anxiety and sleep disorders in older adults: evidence from the 2020 health and retirement study

**DOI:** 10.3389/fpsyg.2026.1810526

**Published:** 2026-05-26

**Authors:** Yang Zhao, Shanna Meng, Zhini Yu, Tao Jiang, Xiangwen Yao, Yanhua Huang, Ying Chen, Na Wang, Zhibin Chen, Lei Zhang

**Affiliations:** 1 School of Humanities and Management, Guilin Medical University, Guilin, Guangxi, China; 2 Guangxi Key Laboratory of Environmental Exposomics and Entire Lifecycle Health, Guangxi Key Laboratory of Diabetic Systems Medicine, School of Public Health, Guilin Medical University, Guilin, Guangxi, China; 3 The First Affiliated Hospital of Guilin Medical University, Guilin, Guangxi, China; 4 Guilin Social Welfare Hospital (Guilin Mental Health Center), Guilin, Guangxi, China; 5 School of Artificial Intelligence Medicine, Guilin Medical University, Guilin, Guangxi, China

**Keywords:** anxiety, life satisfaction, mediation analysis, older adults, self-rated health, sleep disorders

## Abstract

**Background:**

Sleep disorders and anxiety are both common in older adults and are closely related to health and wellbeing. However, evidence from large population-based samples remains limited, particularly regarding the potential roles of self-rated health and life satisfaction in this association.

**Objective:**

To examine the prevalence of anxiety symptoms and sleep disorders in older adults and to investigate the association between anxiety and sleep disorders using data from the 2020 Health and Retirement Study.

**Methods:**

This cross-sectional study used data from the 2020 Health and Retirement Study. A total of 3,436 participants aged 60 years and older who completed the psychosocial questionnaire were included. Chi-square tests, multivariable logistic regression, and mediation analyses were performed to examine the association between anxiety and sleep disorders.

**Results:**

Among the participants, 19.6% had sleep disorders and 12.8% had anxiety symptoms. In the multivariable logistic regression analysis, anxiety was significantly associated with higher odds of sleep disorders (OR = 2.113, 95% CI: 1.677–2.664). Mediation analysis indicated that self-rated health and life satisfaction were statistically significant indirect pathways in this association. The indirect effect through self-rated health was 0.04, accounting for 21.95% of the total association, and the indirect effect through life satisfaction was 0.03, accounting for 17.04%.

**Conclusion:**

In this sample of older adults, anxiety was significantly associated with sleep disorders. Self-rated health and life satisfaction may partly account for this association. Because of the cross-sectional design, the findings should be interpreted as associations rather than causal relationships.

## 1 Introduction

Population aging has become a major public health challenge worldwide. As life expectancy continues to increase, a growing proportion of the population is entering older age, and maintaining health and quality of life in later life has become increasingly important (United Nations, 2022; Chen, 2022; Noren Hooten et al., 2022; Dogra et al., 2022). Among the many issues affecting older adults, sleep problems deserve particular attention. Sleep is closely related to physical functioning, emotional wellbeing, cognitive performance, and daily vitality. Poor sleep in later life is common and may substantially reduce quality of life.

Sleep disorders are frequently reported among older adults, although their determinants are often complex. In addition to chronic disease and lifestyle factors, psychological factors appear to play an important role (Roberts et al., 2000; Xiong et al., 2019; Mookerjee et al., 2023). Previous studies have shown that sleep problems are common in later life, but reported prevalence estimates vary across populations and measurement tools (van de Straat and Bracke, 2015; Wang et al., 2020). This variation makes it important to examine sleep disorders in large population-based datasets using clearly defined measures.

Anxiety is one of the most common psychological symptoms in older adults and has been associated with a range of adverse health outcomes (Baxter et al., 2013; Wang and Pan, 2021; Gould et al., 2016). Older adults with anxiety symptoms may experience persistent worry, nervousness, physical tension, and difficulty relaxing, all of which may be related to difficulty falling asleep, frequent nighttime awakening, early morning awakening, and non-restorative sleep (Magee and Carmin, 2010; Cox and Olatunji, 2020). Prior studies have generally suggested that anxiety symptoms are associated with poorer sleep in older adults, and that more severe anxiety is often accompanied by more severe sleep problems (Yu et al., 2016; Shi et al., 2020). However, much of the available evidence comes from region-specific studies or relatively small samples, which may limit generalizability.

In addition, previous research has mainly focused on whether anxiety is associated with sleep problems, while less attention has been given to factors that may help explain this association in older adults. Self-rated health and life satisfaction may be particularly relevant in this context. Self-rated health reflects an individual’s overall perception of physical and functional wellbeing and is closely related to mental health and quality of life in later life (Verga et al., 2024). Life satisfaction is also an important component of subjective wellbeing and may be associated with both emotional symptoms and sleep-related outcomes. Older adults with higher anxiety may perceive their health more negatively and report lower life satisfaction, and these factors may in turn be related to sleep disorders. Although such pathways are plausible, population-based evidence on these relationships remains limited, especially in studies focusing specifically on older adults.

The Health and Retirement Study provides an opportunity to examine these issues in a large sample of older adults in the United States. Using data from the 2020 Health and Retirement Study, the present study aimed to examine the prevalence of anxiety symptoms and sleep disorders among adults aged 60 years and older and to investigate the association between anxiety and sleep disorders. In addition, this study explored whether self-rated health and life satisfaction might statistically account for part of this association. Given the cross-sectional nature of the data, the findings are intended to clarify patterns of association rather than establish causal relationships.

## 2 Materials and methods

### 2.1 Data source

This was a cross-sectional study based on data from the 2020 Health and Retirement Study (HRS). The HRS is a nationally representative longitudinal survey of adults aged 50 years and older in the United States, conducted by the University of Michigan. The survey is carried out every 2 years and collects extensive information on demographic characteristics, self-rated health, physical functioning, psychosocial factors, and other aspects of aging. In the present study, data from the 2020 wave were used to examine the association between anxiety symptoms and sleep disorders among adults aged 60 years and older. Because the current analysis was based on data collected at a single time point, the findings were interpreted as associations rather than causal relationships.

### 2.2 Study subjects

The study population consisted of participants in the 2020 Health and Retirement Study who were aged 60 years and older and had completed the psychosocial questionnaire. A total of 3,706 participants initially met the inclusion criteria. After data cleaning and preparation of the analytic dataset, 3,436 participants were included in the final analysis.

### 2.3 Study content

The study variables included anxiety symptoms, sleep disorder status, and a range of demographic, behavioral, and health-related covariates, including language, sex, age group, educational level, marital status, smoking status, alcohol consumption, hypertension, diabetes, self-rated health, and life satisfaction. Self-rated health was categorized as good, fair, or poor, and life satisfaction was categorized as satisfied or dissatisfied. Detailed category definitions are presented in Table 1.

**TABLE 1:** Demographic characteristics of older adults in the study.

Variable	Group	N	Proportion (%)
Language	English	3,215	93.6
	Spanish	221	6.4
Gender	Male	1,385	40.3
	Female	2,051	59.7
Age	60∼69	1,560	45.4
	70∼79	1,062	30.9
	80∼	814	23.7
Education level	Junior high or below	190	5.5
	High school	1,305	38.0
	College or above	1,941	56.5
Marital status	Married	1,947	56.7
	Divorced	509	14.8
	Widowed	745	21.7
	Never married	185	5.4
	Other	50	1.5
Smoking	No	3,169	92.2
	Yes	267	7.8
Drinking	No	1,432	41.7
	Yes	2,004	58.3
Hypertension	No	1,171	34.1
	Yes	2,265	65.9
Diabetes	No	2,490	72.5
	Yes	946	27.5
Self-rated health	Good	2,596	75.6
	Fair	690	20.1
Poor	150	4.4
Life satisfaction	Satisfied	3,294	95.9
	Dissatisfied	142	4.1
Anxiety	No	2,997	87.2
	Yes	439	12.8
Sleep disorder	No	2,764	80.4
	Yes	672	19.6
Total		3,436	100.0

#### 2.3.1 Anxiety symptoms

Anxiety symptoms were assessed using a five-item measure derived from the Beck Anxiety Inventory (Beck et al., 1988) and included in the HRS psychosocial questionnaire. The five items assessed whether participants feared that the worst would happen, felt nervous, experienced trembling of the hands, were afraid of dying, and felt dizzy during the past week. Each item was scored on a four-point scale from 1 to 4, with higher scores indicating more frequent anxiety symptoms. The item scores were summed to obtain a total score ranging from 5 to 20. In this study, participants with a total score of 12 or higher were classified as having anxiety symptoms. In the present sample, the five-item scale showed good internal consistency, with a Cronbach’s alpha of 0.81.

#### 2.3.2 Sleep disorders

Sleep disorders were assessed using a modified four-item Jenkins Sleep Scale (Jenkins et al., 1988). Participants were asked how often during the past month they had difficulty falling asleep, woke up during the night and had difficulty falling asleep again, woke up too early and could not fall asleep again, and felt rested when waking up in the morning. Each item was rated using three response categories: most of the time, sometimes, and rarely or never.

For the present analysis, sleep disorder status was defined according to the coding rule used in this study. Participants were classified as having a sleep disorder if they reported most of the time or sometimes on the first three items and sometimes or rarely or never on the item assessing whether they felt rested in the morning. In the present sample, the four-item scale showed acceptable internal consistency, with a Cronbach’s alpha of 0.68.

### 2.4 Statistical analysis

Excel was used for preliminary data organization, and SPSS 23.0 was used for statistical analyses. Categorical variables were presented as numbers and percentages, and differences between groups were examined using chi-square tests. Sleep disorder status was treated as the dependent variable (0 = no, 1 = yes). Variables with *P* < 0.05 in the univariable analyses were entered into the multivariable logistic regression model, and odds ratios (ORs) with 95% confidence intervals (CIs) were reported. Mediation analyses were conducted using Model 4 of the PROCESS macro in SPSS 23.0, with self-rated health and life satisfaction entered as mediators in separate models. A two-sided *P* < 0.05 was considered statistically significant, and exact *P*-values were reported whenever possible.

## 3 Results

### 3.1 Basic characteristics

A total of 3,436 older adults were included in the analysis. Most participants were English speakers (3,215, 93.6%), while 221 (6.4%) were Spanish speakers. There were 1,385 men (40.3%) and 2,051 women (59.7%). The largest age group was 60–69 years (1,560, 45.4%), followed by 70–79 years (1,062, 30.9%) and 80 years or older (814, 23.7%). In terms of education, 1,941 participants (56.5%) had a college degree or above, 1,305 (38.0%) had a high school education, and 190 (5.5%) had a junior high school education or below.

Most participants were married (1,947, 56.7%), followed by widowed (745, 21.7%) and divorced (509, 14.8%). Most did not smoke (3,169, 92.2%), whereas 2,004 (58.3%) reported alcohol consumption. Hypertension was reported by 2,265 participants (65.9%), and diabetes by 946 (27.5%). In addition, 2,596 participants (75.6%) rated their health as good, and 3,294 (95.9%) reported being satisfied with life. Anxiety symptoms were identified in 439 participants (12.8%). Overall, 672 participants (19.6%) had sleep disorders, whereas 2,764 (80.4%) did not. Detailed characteristics are shown in Table 1.

### 3.2 Analysis of sleep events

The Jenkins Sleep Scale was used to assess the frequency of sleep-related problems during the past month. Among the participants, 523 (15.2%) reported having trouble falling asleep most of the time, and 1,202 (35.0%) reported having this problem sometimes. Nighttime awakening was also common: 767 participants (22.3%) reported waking up during the night most of the time, and 1,409 (41.0%) reported this problem sometimes. In addition, 378 participants (11.0%) reported waking up too early and being unable to fall asleep again most of the time, whereas 1,827 (53.2%) reported rarely or never experiencing this problem. Regarding restorative sleep, 1,488 participants (43.3%) reported that they only sometimes or rarely/never felt well rested when they woke up in the morning. Details are shown in Table 2.

**TABLE 2:** The number and composition ratio of sleep items.

Sleep events	N	Composition ratio%
How often did you have trouble falling asleep?		
Most of the time	523	15.2
Sometimes	1,202	35.0
Rarely or never	1,711	49.8
How often did you have trouble waking up during the night?		
Most of the time	767	22.3
Sometimes	1,409	41.0
Rarely or never	1,260	36.7
How often did you wake up too early and have trouble falling asleep again?		
Most of the time	378	11.0
Sometimes	1,231	35.8
Rarely or never	1,827	53.2
When you woke up in the morning, how often did you feel well-rested?		
Most of the time	1,948	56.7
Sometimes	1,058	30.8
Rarely or never	430	12.5

### 3.3 Analysis of anxiety events

The five-item anxiety measure was used to assess the frequency of anxiety symptoms during the past week. Among the participants, 1,737 (50.6%) reported never fearing that the worst would happen, whereas 690 (20.1%) and 82 (2.4%) reported this symptom sometimes and most of the time, respectively. A total of 1,369 participants (39.8%) reported never feeling nervous, while 893 (26.0%) reported feeling nervous sometimes and 95 (2.8%) reported feeling nervous most of the time. For trembling of the hands, 2,505 participants (72.9%) reported never experiencing this symptom, and 74 (2.2%) reported experiencing it most of the time. Regarding fear of dying, 334 participants (9.7%) reported this symptom sometimes and 46 (1.3%) reported it most of the time. In addition, 2,496 participants (72.6%) reported never feeling dizzy, whereas only 22 (0.6%) reported feeling dizzy most of the time. Details are shown in Table 3.

**TABLE 3:** Frequency and proportion of responses to anxiety items.

Anxiety event	N	Composition ratio%
I feared the worst would happen		
Never	1,737	50.6
Almost never	927	27.0
Sometimes	690	20.1
Most of the time	82	2.4
I felt nervous		
Never	1,369	39.8
Almost never	1,079	31.4
Sometimes	893	26.0
Most of the time	95	2.8
My hands were trembling		
Never	2,505	72.9
Almost never	537	15.6
Sometimes	320	9.3
Most of the time	74	2.2
I was afraid of dying		
Never	2,434	70.8
Almost never	622	18.1
Sometimes	334	9.7
Most of the time	46	1.3
I felt dizzy		
Never	2,496	72.6
Almost never	583	17.0
Sometimes	335	9.7
Most of the time	22	0.6

### 3.4 Univariate analysis of factors associated with sleep disorders in older adults

The chi-square test results showed that sleep disorders in older adults were significantly associated with language, sex, educational level, marital status, alcohol consumption, diabetes, self-rated health, life satisfaction, and anxiety symptoms. Specifically, women had a higher proportion of sleep disorders than men (22.2% vs. 15.7%, χ^2^ = 22.314, *P* < 0.001). Participants with lower educational levels had a higher frequency of sleep disorders (χ^2^ = 23.432, *P* < 0.001). Sleep disorders were also more common among those who were divorced, never married, or in the other marital status group than among married participants (χ^2^ = 20.622, *P* < 0.001). In addition, participants who did not consume alcohol, those with diabetes, those with poorer self-rated health, those dissatisfied with life, and those with anxiety symptoms had higher proportions of sleep disorders. By contrast, sleep disorders were not significantly associated with age, smoking status, or hypertension. Details are shown in Table 4.

**TABLE 4:** Univariate analysis of factors associated with sleep disorders in older adults.

Variable	Group	Sleep disorder		Total (%)	χ ^2^	*P*
		No	Yes			
Language	English	2,602 (80.9)	613 (19.1)	3,215 (93.6)	7.652	0.006
	Spanish	162 (73.3)	59 (26.7)	221 (6.4)	-	-
Sex	Male	1,168 (84.3)	217 (15.7)	1,385 (40.3)	22.314	< 0.001
	Female	1,596 (77.8)	455 (22.2)	2,051 (59.7)	-	-
Age (years)	60∼69	1,237 (79.3)	323 (20.7)	1,560 (45.4)	4.880	0.087
	70∼79	851 (80.1)	211 (19.9)	1,062 (30.9)	-	-
	80∼	676 (83.0)	138 (17.0)	814 (23.7)	-	-
Education	Junior high or below	136 (71.6)	54 (28.4)	190 (5.5)	23.432	< 0.001
	High school	1,016 (77.9)	289 (22.1)	1,305 (38.0)	-	-
	College or above	1,612 (83.0)	329 (17.0)	1,941 (56.5)	-	-
Marital status	Married	1,610 (82.7)	337 (17.3)	1,947 (56.7)	20.622	< 0.001
	Divorced	387 (76.0)	122 (24.0)	509 (14.8)	-	-
	Widowed	595 (79.9)	150 (20.1)	745 (21.7)	-	-
	Never married	136 (73.5)	49 (26.5)	185 (5.4)	-	-
	Other	36 (72.0)	14 (28.0)	50 (1.5)	-	-
Smoking	No	2,552 (80.5)	617 (19.5)	3,169 (92.2)	0.200	0.655
	Yes	212 (79.4)	55 (20.6)	267 (7.8)	-	-
Drinking	No	1,116 (77.9)	316 (22.1)	1,432 (41.7)	9.827	0.002
	Yes	1,648 (82.2)	356 (17.8)	2,004 (58.3)	-	-
Hypertension	No	958 (81.8)	213 (18.2)	1,171 (34.1)	2.113	0.146
	Yes	1,806 (79.7)	459 (20.3)	2,265 (65.9)	-	-
Diabetes	No	2,031 (81.6)	459 (18.4)	2,490 (72.5)	7.261	0.007
	Yes	733 (77.5)	213 (22.5)	946 (27.5)	-	-
Self-rated health	Good	2,180 (84.0)	416 (16.0)	2,596 (75.6)	103.435	< 0.001
	Fair	499 (72.3)	191 (27.7)	690 (20.1)	-	-
	Poor	85 (56.7)	65 (43.3)	150 (4.4)	-	-
Life satisfaction	Satisfied	2,669 (81.0)	625 (19.0)	3,294 (95.9)	17.263	< 0.001
	Dissatisfied	95 (66.9)	47 (33.1)	142 (4.1)	-	-
Anxiety	No	2,485 (82.9)	512 (17.1)	2,997 (87.2)	91.249	< 0.001
	Yes	279 (63.6)	160 (36.4)	439 (12.8)	-	-

### 3.5 Multivariate logistic regression analysis of factors influencing sleep disorders

Variables that were statistically significant in the univariable analyses were entered into the multivariable logistic regression model. The results showed that female sex, marital status, self-rated health, and anxiety symptoms were significantly associated with sleep disorders. Specifically, women had higher odds of sleep disorders than men (OR = 1.490, 95% CI: 1.229–1.807, *P* < 0.001). Compared with married participants, divorced participants (OR = 1.289, 95% CI: 1.006–1.650, *P* = 0.045) and never-married participants (OR = 1.528, 95% CI: 1.067–2.189, *P* = 0.021) had higher odds of sleep disorders. In addition, participants with fair self-rated health (OR = 1.610, 95% CI: 1.300–1.994, *P* < 0.001) and poor self-rated health (OR = 2.829, 95% CI: 1.949–4.106, *P* < 0.001) had higher odds of sleep disorders than those with good self-rated health. Anxiety symptoms were also significantly associated with sleep disorders, with an OR of 2.113 (95% CI: 1.677–2.664, *P* < 0.001). Detailed results are shown in Table 5.

**TABLE 5:** Multivariate logistic regression analysis of factors influencing sleep disorders.

Variable	Category	β	S.E.	Wald	P	OR	95% CI	
							Lower limit	Upper limit
Language	English	-	-	-	-	-	-	-
	Spanish	-0.071	0.196	0.131	0.717	0.931	0.634	1.368
Gender	Male	-	-	-	-	-	-	-
	Female	0.399	0.098	16.489	< 0.001	1.490	1.229	1.807
Education level	Junior high or below	-	-	-	-	-	-	-
	High school	-0.093	0.208	0.200	0.655	0.911	0.606	1.371
	College or above	-0.267	0.212	1.579	0.209	0.766	0.505	1.161
Marital status	Married	-	-	-	-	-	-	-
	Divorced	0.254	0.126	4.036	0.045	1.289	1.006	1.650
	Widowed	-0.071	0.119	0.360	0.548	0.931	0.738	1.175
	Never married	0.424	0.183	5.363	0.021	1.528	1.067	2.189
	Other	0.569	0.331	2.948	0.086	1.766	0.923	3.380
Drinking	No	-	-	-	-	-	-	-
	Yes	-0.046	0.093	0.248	0.619	0.955	0.796	1.146
Diabetes	No	-	-	-	-	-	-	-
	Yes	0.125	0.099	1.602	0.206	1.133	0.934	1.375
Self-rated health	Good	-	-	-	-	-	-	-
	Fair	0.476	0.109	19.002	< 0.001	1.610	1.300	1.994
	Poor	1.040	0.190	29.947	< 0.001	2.829	1.949	4.106
Life satisfaction	Satisfied	-	-	-	-	-	-	-
	Dissatisfied	0.085	0.203	0.175	0.676	1.088	0.732	1.619
Anxiety	No	-	-	-	-	-	-	-
	Yes	0.748	0.118	40.147	< 0.001	2.113	1.677	2.664

Reference categories were English for language, male for sex, junior high school or below for educational level, married for marital status, no alcohol consumption for drinking status, no diabetes for diabetes status, good for self-rated health, satisfied for life satisfaction, and no anxiety symptoms for anxiety.

### 3.6 The statistical mediation of self-rated health and life satisfaction in the association between anxiety and sleep disorders

To further explore whether self-rated health and life satisfaction statistically accounted for part of the association between anxiety and sleep disorders, mediation analyses were conducted with these two variables entered separately as mediators.

**FIGURE 1:**
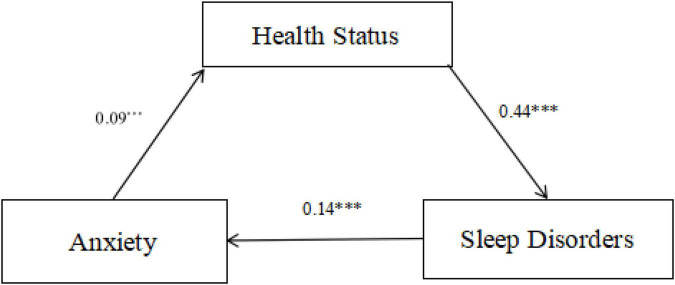
Model 1 path coefficient diagram of Self-rated health in anxiety and sleep disorders. The figure shows the path model with coefficients for anxiety- > Self-rated health, Self-rated health- > sleep disorders, and anxiety- > sleep disorders. *** denotes statistical significance at the *p* < 0.001 level.

In Model 1, anxiety was significantly associated with sleep disorders, with a total effect of 0.18. Anxiety was also significantly associated with self-rated health. After self-rated health was included in the model, the direct effect of anxiety on sleep disorders remained significant at 0.14. The indirect effect through self-rated health was 0.04 (95% CI: 0.03–0.05), accounting for 21.95% of the total association. Detailed results are shown in Figure 1 and Table 6.

**TABLE 6:** Model 1: decomposition of total, direct, and mediating effects (self-rated health as mediator).

Effect	Effect value	SE	LLCI	ULCI	Effect proportion (%)
Total effect	0.18	0.01	0.16	0.20	
Direct effect	0.14	0.01	0.12	0.16	78.05
Mediation effect	0.04	0.00	0.03	0.05	21.95

**FIGURE 2:**
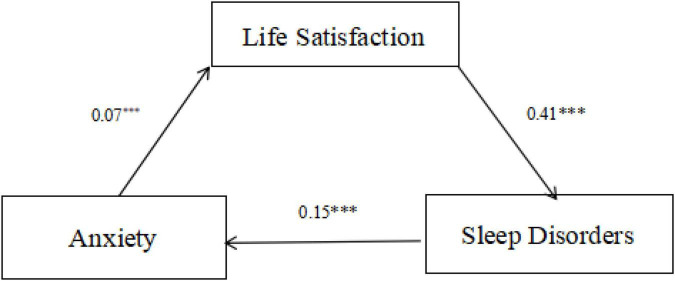
Model 2: Path coefficient diagram of life satisfaction in anxiety and sleep disorders. The figure shows the path model with coefficients for anxiety- > life satisfaction, life satisfaction- > sleep disorders, and anxiety- > sleep disorders. *** denotes statistical significance at the *p* < 0.001 level.

In Model 2, anxiety was significantly associated with sleep disorders, with a total effect of 0.18. Anxiety was also significantly associated with life satisfaction. After life satisfaction was included in the model, the direct effect of anxiety on sleep disorders remained significant at 0.15. The indirect effect through life satisfaction was 0.03 (95% CI: 0.02–0.04), accounting for 17.04% of the total association. Detailed results are shown in Figure 2 and Table 7.

**TABLE 7:** Model 2: decomposition of total, direct, and mediating effects (life satisfaction as mediator).

Effect	Effect value	SE	LLCI	ULCI	Effect proportion (%)
Total effect	0.18	0.01	0.16	0.20	
Direct effect	0.15	0.01	0.13	0.17	82.96
Mediation effect	0.03	0.00	0.02	0.04	17.04

Overall, these findings suggest that self-rated health and life satisfaction may partly account for the observed association between anxiety and sleep disorders in older adults. Given the cross-sectional design of the study, these results should be interpreted as statistical mediation findings rather than evidence of causal pathways.

## 4 Discussion

### 4.1 Current status of sleep disorders in older adults

In the present study, 19.6% of older adults were classified as having sleep disorders. This prevalence is similar to that reported in some community-based studies of older populations, but lower than the estimates reported in several studies conducted in China and Europe (Wang and Su, 2021; Liu et al., 2014; van de Straat and Bracke, 2015). One possible explanation for this difference is variation in the measurement tools used across studies. In the present study, sleep disorders were assessed using a modified Jenkins Sleep Scale, and differences in measurement tools across studies may have contributed to the variation in prevalence estimates. Differences in study population characteristics, sampling strategies, cultural background, and analytic methods may also have contributed to the variation in prevalence estimates.

These findings suggest that sleep problems are common among older adults and remain an important public health concern. Even though the prevalence observed in this study was lower than that reported in some previous studies, nearly one in five participants had sleep disorders. This highlights the importance of continued attention to sleep health in older adults, especially in large population-based studies that allow the relationship between sleep and psychological factors to be examined in a broader context.

### 4.2 Factors influencing sleep disorders in older adults

#### 4.2.1 Gender

In the present study, the prevalence of sleep disorders was higher in women than in men, and this association remained statistically significant in the multivariable analysis. Women had higher odds of sleep disorders than men, with an OR of 1.49. This finding is consistent with previous studies showing that sleep problems are more common among women than among men (Wang et al., 2019; Lin et al., 2008). One possible explanation is that hormonal changes after menopause may contribute to poorer sleep in women (Xu et al., 2010). Social and psychological factors may also play a role. Compared with men, older women may experience different life stressors, caregiving burdens, and patterns of emotional distress, which may be related to sleep problems. These findings suggest that sex differences should be considered when developing strategies to improve sleep health in older adults.

#### 4.2.2 Anxiety

In the present study, anxiety symptoms were significantly associated with sleep disorders in older adults. Participants with anxiety symptoms had higher odds of sleep disorders than those without anxiety symptoms, even after adjustment for sex, educational level, marital status, alcohol consumption, diabetes, self-rated health, and life satisfaction. This finding is consistent with previous studies showing that anxiety is closely related to sleep problems in older adults (Cox and Olatunji, 2020; Yu et al., 2016; Magee and Carmin, 2010; Shi et al., 2020). One possible explanation is that anxiety symptoms, such as excessive worry, nervousness, and physiological arousal, may be associated with difficulty falling asleep, frequent nighttime awakening, and poorer overall sleep quality (Cox and Olatunji, 2020; Magee and Carmin, 2010). At the same time, sleep problems may also exacerbate emotional distress. Given the cross-sectional design of the present study, the results should therefore be interpreted as an association rather than evidence that anxiety causes sleep disorders. These findings suggest that anxiety symptoms should be considered when evaluating sleep health in older adults.

### 4.3 Mediation effect model analysis

The mediation analyses showed that self-rated health and life satisfaction each accounted for part of the association between anxiety and sleep disorders in older adults. Specifically, the indirect effect through self-rated health was 0.04, accounting for 21.95% of the total association, whereas the indirect effect through life satisfaction was 0.03, accounting for 17.04% of the total association. These findings suggest that the association between anxiety and sleep disorders may partly be linked to differences in perceived health and subjective wellbeing.

This interpretation is broadly consistent with previous studies showing that sleep is closely related to life satisfaction in older adults (Papi and Cheraghi, 2021; Zhi et al., 2016). In addition, self-rated health has also been shown to be strongly associated with sleep quality (Hublin et al., 2018). Mediation-based research has further suggested that sleep quality, mental health, and life satisfaction may be linked through indirect pathways (Wang et al., 2023).

At the same time, these results should be interpreted with caution. Because the present study was based on cross-sectional data, the mediation findings reflect statistical pathways rather than confirmed causal mechanisms. Future longitudinal studies are needed to clarify the temporal order of anxiety, self-rated health, life satisfaction, and sleep disorders in older adults.

#### 4.3.1 Limitations of the study

This study has several limitations. First, because the analysis was based on cross-sectional data from the 2020 Health and Retirement Study, the temporal order of anxiety symptoms, self-rated health, life satisfaction, and sleep disorders could not be established. Therefore, the findings should be interpreted as associations rather than evidence of causality. Second, although multiple covariates were included in the analyses, some potentially important factors, such as depressive symptoms, pain, and socioeconomic characteristics, were not included. Residual confounding cannot therefore be excluded. Third, the main study variables were derived from self-reported questionnaire data, which may have been affected by recall bias or reporting bias. Fourth, the analytic sample was based on participants with available data on the study variables, and detailed reasons for all exclusions from the initial eligible sample were not fully documented in the archived analytic materials. This may have affected the transparency of sample selection. Finally, multicollinearity diagnostics were not formally performed in the present analysis, and possible collinearity among covariates cannot be completely ruled out. Future studies should use longitudinal designs, include a broader range of psychosocial and health-related variables, and further assess model assumptions to provide more robust evidence on the association between anxiety and sleep disorders in older adults.

#### 4.3.2 Strengths of the study

This study has several strengths. First, it used data from the 2020 Health and Retirement Study, which provided a relatively large sample of older adults and allowed the association between anxiety and sleep disorders to be examined in a population-based dataset. Second, the study assessed anxiety symptoms and sleep-related problems using structured questionnaire measures and showed acceptable internal consistency in the analytic sample. Third, beyond examining the overall association between anxiety and sleep disorders, the study further explored the potential roles of self-rated health and life satisfaction, which adds to the interpretation of the observed findings. Finally, multiple demographic and health-related variables were included in the analyses, which helped provide a more comprehensive assessment of factors associated with sleep disorders in older adults.

## 5 Conclusion

Using data from the 2020 Health and Retirement Study, this study found that anxiety symptoms were significantly associated with sleep disorders among adults aged 60 years and older. Self-rated health and life satisfaction may partly account for this association. Because of the cross-sectional design, these findings should be interpreted as associations rather than evidence of causality. Overall, the results highlight the importance of considering psychological wellbeing, perceived health, and subjective wellbeing when evaluating sleep health in older adults.

## Data Availability

The original contributions presented in the study are included in the article/supplementary material, further inquiries can be directed to the corresponding authors.
